# Association of Daily Step Count With Depressive Symptoms in Patients With Major Depressive Disorder Using a Smartphone App (ReMAP): Longitudinal Study

**DOI:** 10.2196/81120

**Published:** 2026-02-10

**Authors:** Alexander Refisch, Lara Gutfleisch, Daniel Emden, Vincent Holstein, Marius Gruber, Janik Goltermann, Maike Richter, Janette Ratzsch, Anna Fleuchhaus, Elisabeth Leehr, Susanne Meinert, Tiana Borgers, Kira Flinkenfügel, Frederike Stein, Florian Thomas-Odenthal, Paula Usemann, Lea Teutenberg, Nina Alexander, Ronny Redlich, Igor Nenadić, Tilo Kircher, Udo Dannlowski, Tim Hahn, Nils Opel

**Affiliations:** 1 Department of Psychiatry and Psychotherapy, Jena University Hospital, Jena, Germany; 2 Department of Psychiatry, Psychotherapy and Psychosomatics, Catholic Hospital “St. Johann Nepomuk”, Erfurt, Germany; 3 Department of Psychiatry and Neuroscience, Charite - Universitätsmedizin Berlin, Campus Benjamin Franklin, Berlin, Germany; 4 Institute for Translational Psychiatry, University of Münster, Münster, Germany; 5 McLean Hospital, Belmont, MA, United States; 6 Department of Psychiatry, Harvard Medical School, Boston, MA, United States; 7 Stanley Center for Psychiatric Research, Broad Institute of MIT and Harvard, Cambridge, MA, United States; 8 Department of Psychiatry, Psychosomatic Medicine, and Psychotherapy, Goethe University Frankfurt, University Hospital, Frankfurt, Germany; 9 Cooperative Brain Imaging Center-CoBIC, Goethe University Frankfurt, Frankfurt, Germany; 10 Department of Clinical Psychology and Psychotherapy, University of Göttingen, Göttingen, Germany; 11 Institute for Translational Neuroscience, University of Münster, Münster, Germany; 12 Department of Psychiatry and Psychotherapy, University of Marburg, Marburg, Germany; 13 Department of Psychology, University of Halle, Halle, Germany; 14 German Center for Mental Health (DZPG), Halle-Jena-Magdeburg, Germany; 15 Center for Intervention and Research on Adaptive and Maladaptive Brain Circuits Underlying Mental Health (C-I-R-C), Jena Magdeburg Halle, Germany; 16 Center for Mind, Brain and Behavior (CMBB), University of Marburg, Marburg, Germany; 17 Department of Psychiatry, Medical School and University Medical Center OWL, Protestant Hospital of the Bethel Foundation, Bielefeld University, Bielefeld, Germany; 18 Department of Neurology, Jena University Hospital, Am Klinikum 1, Jena, 07747, Germany; 19 German Center for Mental Health (DZPG), partner site Berlin-Potsdam, Berlin, Germany

**Keywords:** depression, physical activity, mood, smartphone, mobile phone, application, app

## Abstract

**Background:**

The benefits of physical activity (PA) for both physical and mental health, including major depressive disorder (MDD), are well established. Mobile devices, such as smartphones, offer a scalable way to monitor PA and its relationship with depressive symptoms in daily life.

**Objective:**

This study aimed to investigate the association between passive smartphone-recorded step counts and current depressive symptoms in individuals with and without a lifetime diagnosis of MDD, using a naturalistic bring-your-own-device approach.

**Methods:**

We used the Remote Monitoring Application in Psychiatry (ReMAP) to collect passive step count data from participants’ personal smartphones. The sample included 181 individuals with a lifetime MDD diagnosis, assessed via the structured clinical interview for the *Diagnostic and Statistical Manual of Mental Disorders* (Fourth Edition; *DSM-IV*), and 195 healthy controls (HCs). Current depressive symptoms were assessed using the Beck Depression Inventory. PA was operationalized as daily and weekly step counts, passively recorded via smartphone sensors. Hierarchical models were applied to examine the association between PA and depression severity.

**Results:**

Patients with MDD exhibited significantly lower daily step counts (mean 3454, SD 2683) compared to HCs (mean 4699, SD 3175; *P*<.001) and showed reduced diurnal variability (β=–0.36; *P*=.003). Higher daily step counts were associated with lower Beck Depression Inventory scores across the full sample (β=–0.06, 95% CI –0.09 to –0.02; *P*=.002), with similar trends in both MDD and HC groups. Weekly step counts also significantly predicted lower concurrent depressive symptoms (β=–0.29, 95% CI –0.43 to –0.14; *P*<.001), while patients with MDD displayed less variability in weekly activity levels than HCs (β=–0.75; *P*=.001).

**Conclusions:**

These findings underscore the potential of mobile devices to be used as effective tools for monitoring PA in patients with MDD, supporting more customized and adaptive approaches to prevention and treatment. They also emphasize the importance of incorporating PA into the clinical management of depression.

## Introduction

Major depressive disorder (MDD) is a significant public health concern that affects 15%‐18% of individuals over their lifetime [1], resulting in impaired psychosocial functioning, reduced quality of life, and shortened life expectancy [2,3].

Available treatment options are far from optimal, with only about one-third of patients responding to antidepressant medication as a first-line treatment for depression [4]. While psychotherapy represents another recommended first-line approach, response rates remain limited, and access barriers persist [5]. Importantly, psychotherapy may offer a more suitable context to integrate and promote adjunctive interventions, such as physical activity (PA), which is increasingly recognized as an effective component in the treatment of MDD.

The antidepressant effects of PA are well supported by meta-analyses, showing moderate to large effect sizes, though randomized controlled trials differ considerably in sample size, exercise type, and control conditions [6]. While the beneficial effects of PA on depressive symptoms are well established, the intensity and duration of exercise that is most effective for the treatment and prevention of MDD remain controversial [7]. Importantly, augmenting standard treatments with exercise has shown improved outcomes and minimal risk of adverse effects [8], and prospective data indicate that even subthreshold levels are associated with a reduced risk of developing MDD [9].

PA modulates key biological processes implicated in depression, including immune function, neuroplasticity, and the hypothalamic-pituitary-adrenal axis [10,11], and may exert antidepressant effects partly through reducing low-grade inflammation, a shared feature of metabolic disorders and MDD [12-14]. Despite the compelling evidence presented, adherence to PA in the real world remains a challenge, and there is a paucity of knowledge regarding the relationship between naturally occurring daily activity and depressive symptoms outside of structured interventions.

Step counting is a widely adopted and popular metric for quantifying PA in both research and the general population [15-21]. The proliferation of wearable devices and smartphone apps has made step counting accessible and intuitive, facilitating its use for self-monitoring and goal setting across diverse populations. The American College of Sports Medicine recognizes daily step counts as an easily interpretable unit of locomotion increasingly used in public health recommendations, research, and clinical practice [15,22]. This quantification is typically performed via smartphones and wearables. There is growing evidence that links step counts to physical and mental health outcomes, including MDD [23,24]. Walking, as the most prevalent form of PA, presents a strategy that is characterized by its low-barrier nature and scalability, thereby accommodating individual needs [25]. Step counts also provide an interpretable and goal-oriented measure of overall activity, capturing not only deliberate exercise but also habitual movement in daily life [26,27].

However, the majority of prior research has relied on controlled devices or structured interventions. In contrast, the present study addresses a significant research gap by examining the relationship between depressive symptoms and step counts in a real-world, naturalistic setting using participants’ own smartphones; a bring-your-own-device (BYOD) approach. While there is variability in the accuracy of commercial sensors such as wearables [28,29], smartphone-based step counts have demonstrated reasonable validity under everyday conditions [30,31]. Consequently, BYOD monitoring emerges as a pragmatic and scalable instrument for large-scale mental health research.

However, it remains unclear whether the antidepressant effects of PA are primarily driven by the total number of steps per day or by specific patterns of activity, such as daily variability or potential threshold effects. For example, it is not yet known whether very high activity levels may also be associated with negative outcomes. Using smartphone-based data allows us to explore such nuanced real-world activity patterns in greater detail.

Accordingly, the present study investigates the association between daily step counts and self-reported depressive symptoms using Remote Monitoring Application in Psychiatry (ReMAP), which enables continuous, passive behavioral monitoring with high temporal resolution and validated symptom assessments [32,33]. Given the substantial evidence supporting PA as an effective treatment and preventative measure for depressive symptoms, our primary hypothesis posits that patients with MDD exhibit lower PA levels, operationalized as daily step counts, compared to healthy controls (HCs). Second, we hypothesize that higher step counts are associated with lower depression severity, as reflected in Beck depression inventory (BDI) scores. Finally, we aim to explore whether variability in step counts across the week is related to depression severity, including the possibility that both low and excessively high activity levels may be associated with worse outcomes.

## Methods

### Study Design

This study used a naturalistic, prospective, and longitudinal cohort design. Participants with MDD and HCs were followed over time through smartphone-based assessments of PA (daily step counts) and depressive symptoms (repeated BDI assessments). This design allowed for the observation of within-person variation and between-group differences under real-world conditions.

### Participants

The sample included 181 participants with a lifetime diagnosis of MDD and 195 HCs, resulting in a total of 376 participants with available data from ReMAP. MDD diagnoses were established based on the structured clinical interview for the *Diagnostic and Statistical Manual of Mental Disorders, Fourth Edition* (SCID) and verified clinical records. An acute depressive episode was not required for inclusion, as the study focused on lifetime MDD status and current variation in depressive symptom severity.

Participants were recruited from several ongoing longitudinal clinical cohorts: the Marburg/Münster Affective Disorder Cohort Study (n=47) [34], the Münster Neuroimage Cohort (n=17) [35,36], 2 subsamples of the SFB-TRR58 cohort (n=81; Z02 Münster and SpiderVR Münster) [37], and the TIP cohort (n=28). These cohorts include both HC participants and patient groups currently or formerly receiving inpatient treatment for affective disorders, ensuring diagnostic validity through structured assessment and clinical verification. The presence of a current or lifetime diagnosis of mental disorders was assessed using SCID interviews in all participants [38]. HC participants were confirmed to be free of any current or past psychiatric disorders based on SCID interviews. Participants with severe physical or neurological conditions (eg, cardiovascular, oncological, or neurodegenerative diseases) were excluded during recruitment in all contributing cohorts to minimize potential health-related confounding.

Depressive symptoms were assessed using the 21-item BDI [26], a validated self-report measure of depressive symptom severity (total score range 0‐63 and higher scores indicating greater severity). For the present analyses, BDI total scores were treated as continuous variables to capture dimensional variation in depressive symptom burden rather than categorized into discrete severity levels. Participants were required to complete at least 3 BDI assessments, each separated by a minimum of 1 week, to capture within-subject fluctuations in depressive symptoms in relation to PA.

For step count data, days exceeding 50,000 steps were excluded to account for implausible or erroneous values.

The final sample (n=376; 181 MDD, 195 HCs) included all participants meeting the inclusion criteria and providing sufficient data for both step counts and BDI assessments. No a priori sample size calculation was performed because this study used existing data from several ongoing longitudinal cohorts. To assess statistical sensitivity, a post hoc power analysis using G*Power (v3.1; Heinrich Heine University Düsseldorf, Germany) was conducted. Assuming an *α* of .05, power of 0.80, and 8 predictors in a linear model approximation, the minimal detectable effect was f^2^=0.052, indicating adequate power to detect small-to-medium effects commonly observed in PA and depression research.

### ReMAP

ReMAP is a smartphone-based monitoring app developed at the University of Münster [32]. Following a BYOD approach, it passively records step counts and distance via Apple Health or Google Fit and collects active self-reports such as the BDI at regular intervals. All data are pseudonymized, encrypted, and stored in compliance with data protection regulations. The validity of ReMAP-derived measures has been demonstrated in previous studies [33].

### Depression

Depressive symptoms are measured with the BDI, consisting of 21 items [39]. Both self-report and clinician-rated evaluations have been shown to be consistent, affirming the reliability of smartphone-based self-report measures in assessing depressive symptoms [33]. To evaluate convergent validity in the present sample, ReMAP-derived BDI (digital) scores were compared with the paper-pencil BDI and the clinician-rated Hamilton Depression Rating Scale (HAMD) at baseline using Pearson correlations and intra-class correlation (ICC; 2-way, absolute agreement, single measure).

### Physical Activity

PA was measured as daily step counts using the ReMAP app. Pedometers and accelerometers in phones generate step count data and distance information. Android phones also use GPS locations to better estimate distance [32].

To address the substantial variability in step data, both in daily step counts and in day-to-day variability, we adopted the activity level classification framework proposed by Tudor-Locke and Bassett. This framework defines 3 categories based on established PA guidelines: sedentary (≤4999 steps/d), moderate (5000‐9999 steps/d), and high (>9999 steps/d) PA levels [19]. These categories, along with total step counts, were used to characterize PA levels in subsequent analyses. To account for differences in assessment timing, we focused the analysis on the week surrounding each BDI measurement.

### Statistical Analysis

#### Comparative Analysis of PA Levels Between Patients With MDD and HC

We conducted a descriptive analysis of PA levels categorized as sedentary (≤4999 steps/d), moderate (5000‐9999 steps/d), and high (>9999 steps/d) for patients with MDD and HCs [19]. Chi-square tests were used to assess significant differences in baseline PA levels between diagnostic groups.

#### Association Between PA Levels and Depression Symptom Severity

To examine the relationship between PA and depressive symptom severity, we conducted linear mixed-effects models with random intercepts for each participant. The BDI scores served as the dependent variable, analyzed both in the overall sample and separately within the MDD and HC groups. Missing step count data were handled with a mixed linear model approach. For each BDI measurement, step count data within a 3-day window were considered for analysis. Given the naturalistic study design and varying numbers of valid step count days and assessment intervals across participants, linear mixed effects models were applied to account for the unbalanced data structure and to include all available observations per participant that met the inclusion and exclusion criteria. We assessed the impact of both peak daily step counts within a week and cumulative weekly step totals. In one model, PA was entered as a categorical variable (sedentary, moderate, and high), while in another, daily and weekly step counts were treated as continuous predictors. For interpretability, the sum of daily and weekly step counts was z-standardized prior to modeling, so that coefficients represent the change in depressive symptom scores per one standard deviation change in step counts.

In patients with MDD, psychotropic medication use was statistically controlled for by including a composite medication index representing the number and type of psychotropic medications as an additional covariate in the linear mixed-effects models.

Covariates were selected based on prior evidence of factors influencing both PA and depressive symptom reporting in smartphone-based monitoring studies. Age, gender, and smartphone platform (iOS vs Android) were included to control for demographic and technical variability. In a sensitivity analysis, BMI was additionally included in the model to assess the potential influence of physical health status.

#### Variability in PA and Its Relation to Depression Severity

As there are currently no established metrics for short-term variability in daily PA levels in the context of MDD, we conducted an exploratory analysis to quantify and compare intra-week variability patterns. In total, 2 complementary variables were derived from the data set listed below:

1. Weekly PA level variability: Each participant’s daily step count was categorized into one of 3 PA levels: sedentary, moderate, or high, based on established cutoffs. The weekly variability score represents the number of distinct PA levels reached within a week (range=1‐3). A value of 1 indicates that all days fell within the same PA level (no variability), whereas 3 indicates that all 3 PA levels occurred within the same week (maximum variability). This metric was designed to capture whether individuals who fluctuate between different activity levels during a week differ in depressive symptom severity compared to those with stable activity patterns.
2. Daily PA level variability: This variable quantifies the number of transitions (shifts) between PA levels across consecutive days within a week (range =0‐6). A score of 0 indicates complete stability (no day-to-day change in PA level), and 6 indicates the maximum variability, with a change in PA level on every day of the week. This measure captures short-term instability in PA behavior.

First, a cumulative log model was performed to analyze ordinal weekly variability and to differentiate between diagnostic groups (MDD vs HC). Second, a general linear mixed-effects model with Poisson distribution was used to look at daily differences in diagnostic groups (MDD vs HC). In both models, a random intercept was added to account for repeated measurements for each participant. The HC group was used as the reference category.

### Ethical Considerations

The study was reviewed and approved by the Ethics Committee of the Medical Association of Westphalia-Lippe and the Westphalian Wilhelms University of Münster (approval number 2017-615-f-S). All participants provided written informed consent prior to participation. Data were collected and stored in pseudonymized form in accordance with the General Data Protection Regulation. To acknowledge their time and effort, participants received a modest compensation of €20 (approximately US $22).

## Results

### Study Sample

**Figure 1.**
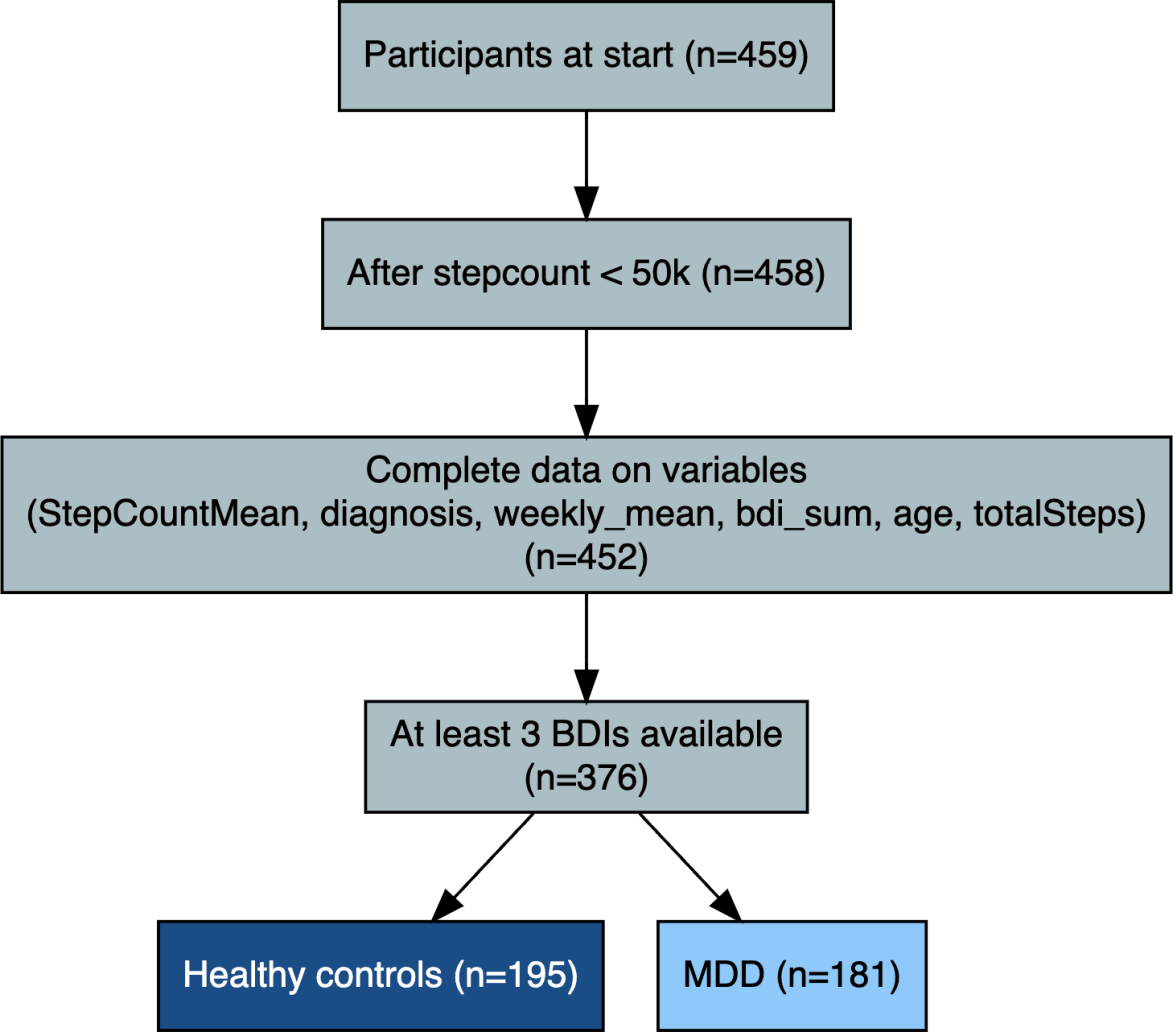
Participant flow diagram following STROBE (Strengthening the Reporting of Observational Studies in Epidemiology) recommendations, showing the number of individuals screened, excluded (with reasons), and included in the final analyses for healthy controls and participants with major depressive disorder. BDI: Beck Depression Inventory; MDD: major depressive disorder.

The inclusion process and participant numbers at each stage are summarized in Figure 1. The study included 376 participants with a mean age of 44.76 (SD 13.83) years, of whom 268 were female (Table 1). Of these, 181 participants met diagnostic criteria for lifetime MDD based on the SCID. The mean BDI score for the entire cohort was 7.36 (SD 9.24), and participants reported a mean number of daily steps of 4113 (SD 3019). Patients with MDD showed significantly lower mean daily step counts than HC (3454, SD 2683 vs 4699, SD 3175 steps/day; *P*<.001). ReMAP BDI scores showed moderate-to-strong convergence with conventional assessments. Specifically, correlations with paper-pencil BDI were *r*=0.77 (ICC=0.67, 95% CI 0.45‐0.81) and with Hamilton Depression Rating Scale, *r*=0.57 (ICC=0.61, 95% CI 0.36‐0.78). These results confirm adequate convergent validity of the digital ReMAP BDI in the current sample, albeit with slightly lower coefficients compared to those reported by Goltermann et al [33]. In an additional sensitivity analysis including BMI as a covariate, BMI did not significantly predict depressive symptom severity (*β*=.007; *P*=.84), and the association between step counts and depressive symptoms remained unchanged.

**Table 1.** Sociodemographic characteristics and comparison between patients with MDD and healthy controls during the study period.

Variables	Participants with MDD^a^ (n=181)	Healthy controls (n=195)
Age (years), mean (SD)	44.22 (12.89)	45.24 (14.60)
Sex, n		
Male	51	57
Female	130	138
Living situation, n (%)^b^		
Alone	34 (23.8)	27 (18.6)
Partner	50 (35.6)	66 (46)
Shared	17 (11.9)	20 (13.7)
Other	41 (28.7)	31 (21.7)
Occupational status, n (%)^b^		
Full-time	49 (34.2)	60 (41.9)
Part-time	30 (21.5)	36 (25)
Unemployed	12 (8.2)	1 (0.6)
Training	25 (17.7)	29 (20)
Other	26 (18.4)	18 (12.5)
Education (years), mean (SD)^b^	13.6 (2.85)	14.3 (2.79)
Disposable household income (US $/month), mean (SD)^b^	2978 (1980)	3622 (2287)
BMI, mean (SD)^b^	27.3 (6.51)	25.1 (5.03)
BDI^c^, mean (SD)	12.42 (10.85)	2.86 (3.70)
Step counts per day, mean (SD)	3454 (2683)	4699 (3175)

a MDD: major depressive disorder.

b Data on living situation, occupational status, education, disposable household income, and BMI were available for a subsample (MDD: n=142; healthy controls: n=144). Percentages and means are based on the available data.

c BDI: Beck Depression Inventory.

### Comparative Analysis of PA Levels Between Patients With MDD and HC

Chi-square tests revealed significant differences in the distribution of baseline PA levels between patients with MDD and HC (*χ*^2^_2_=8.16; *P*=.02). Patients with MDD were more likely to exhibit sedentary PA levels (75/181, 41.4%) compared to HCs (55/195, 28.2%), whereas HCs were more likely to exhibit high PA levels (64/195, 32.8%) than patients with MDD (42/181, 23.2%). The moderate PA category included 35.4% (64/181) of patients with MDD and 39% (76/195) of HCs. The distribution of steps per day can be viewed in Multimedia Appendix 1.

### Association Between PA Levels and Depression Symptom Severity

Higher PA levels, both categorical (b=–0.37; 95% CI –0.51 to –0.22; *P*<.001) and total daily step counts (b=–.06; 95%CI –0.09 to –0.02; *P*=.002), were significantly associated with lower BDI scores in the whole sample as well as in patients with MDD and HC (Tables2  3).

**Table 2.** Linear mixed model of daily step count categories and depressive symptoms (Beck depression inventory).

Variables	Whole sample			MDD^a^			HC^b^		
	Estimate (95% CI)		*P* value	Estimate (95% CI)		*P* value	Estimate (95% CI)		*P* value
Intercept	1.26 (1.60 to 4.11)		.39	11.49 (5.95 to 17.04)		<.001^c^	1.87 (0.18 to 3.55)		.03^d^
Moderate physical activity	–0.04 (–0.14 to 0.06)		.46	–0.10 (–0.29 to 0.10)		.33	–0.01 (–0.07 to 0.09)		.84
High physical activity	–0.37 (–0.51 to –0.22)		<.001^c^	–0.52 (–0.83 to –0.21)		<.001^c^	–0.28 (–0.39 to –0.17)		<.001^c^
Age	0.01 (–0.04 to 0.07)		.60	0.02 (–0.09 to 0.14)		.73	0.01 (–0.02 to 0.04)		.55
Gender (women)	0.72 (–0.99 to 2.43)		.41	1.01 (–2.37 to 4.4)		.56	0.38 (–0.65 to 1.41)		.47
Platform (Android)	1.13 (–0.17 to 2.44)		.09	1.26 (–0.92 to 3.47)		.26	0.68 (–0.25 to 1.6)		.15
Diagnosis (MDD)	10.73 (9.18 to 12.27)		<.001^c^	—^e^		—	—		—

a MDD: major depressive disorder.

b HC: healthy control.

c *P*<.001

d *P*<.05.

e Not available.

**Table 3.** Linear mixed model of z-standardized daily step counts and depressive symptoms (Beck Depression Inventory).

Variables	Whole sample			MDD^a^			HC^b^		
	Estimate (95% CI)		*P* value	Estimate (95% CI)		*P* value	Estimate (95% CI)		*P* value
Intercept	1.16 (–1.70 to 4.02)		.43	11.36 (5.81 to 16.90)		<.001^c^	1.81 (0.13 to 3.50)		.04^d^
Step count sum (z-standardized daily step counts)	–0.06 (–0.09 to –0.02)		.002^e^	–0.06 (–0.13 to 0.003)		.06	–0.05 (–0.08 to –0.02)		.01^d^
Age	0.02 (–0.04 to 0.07)		.59	0.02 (–0.09 to 0.14)		.72	0.01 (–0.02 to 0.04)		.53
Gender (women)	0.73 (–0.97 to 2.44)		.40	1.05 (–2.34 to 4.43)		.55	0.39 (–0.65 to 1.43)		.46
Platform (Android)	1.15 (–0.15 to 2.44)		.08	1.29 (–0.90 to 3.50)		.25	0.69 (–0.24 to 1.63)		.15
Diagnosis (MDD)	10.74 (9.19 to 12.29)		<.001^c^	—^f^		—	—		—

a MDD: major depressive disorder.

b HC: healthy controls.

c *P*<.001

d *P*<.05

e *P*<.01

f Not available.

Analyses of PA patterns over an entire week revealed that weekly step counts were associated with lower BDI scores (b=–0.29, 95% CI –.43 to –.14; *P*<.001) within diagnostic groups (Table 4), while no significant association was observed for categorical step count levels (Table 5).

**Table 4.** Linear mixed model of z-standardized weekly step counts and depressive symptoms (Beck Depression Inventory).

Variables	Whole sample			MDD^a^			HC^b^		
	Estimate (95% CI)		*P* value	Estimate (95% CI)		*P* value	Estimate (95% CI)		*P* value
Intercept	1.30 (–1.55 to 4.16)		.37	11.13 (5.59 to 16.68)		<.001^c^	2.08 (0.38 to 3.78)		.02^d^
Step count sum (z)	–0.29 (–0.43 to –0.14)		<.001^c^	–0.37 (–0.64 to –0.10)		<.001^c^	-0.23 (–0.35 to -0.12)		<.001^c^
Age	0.01 (–0.04 to 0.67)		.70	0.02 (–0.10 to 0.13)		.78	0.01 (–0.03 to 0.04)		.67
Gender (women)	0.55 (–1.15 to 2.25)		.52	0.65 (–2.70 to 4)		.71	0.28 (–0.76 to 1.33)		.60
Platform (Android)	1.69 (0.23 to 3.15)		.02^d^	2.53 (–0.17 to 5.23)		.07	0.61 (–0.34 to 1.55)		.21
Diagnosis (MDD)	10.54 (9 to 12.07)		<.001^c^	—^e^		—	—		—

a MDD: major depressive disorder.

b HC: healthy control.

c *P*<.001

d *P*<.05

e Not available.

**Table 5.** Linear mixed model of weekly step count categories and depressive symptoms (Beck Depression Inventory).

Variables	Whole sample			MDD^a^			HC^b^		
	Estimate (95% CI)		*P* value	Estimate (95% CI)		*P* value	Estimate (95% CI)		*P* value
Intercept	1.07 (–1.79 to 3.95)		.46	10.75 (5.18 to 16.32)		<.001^c^	1.94 (0.23 to 3.66)		.03^d^
Moderate PA^e^	0.13 (–0.14 to 0.41)		.34	0.20 (–0.30 to 0.70)		.43	0.08 (–0.16 to 0.32)		.51
High PA	–0.27 (–0.60 to 0.05)		.10	–0.48 (–1.10 to 0.13)		.12	–0.14 (–0.42 to 0.13)		.30
Age (years)	0.01 (–0.04 to 0.07)		.62	0.02 (–0.09 to 0.14)		.71	0.01 (–0.02 to 0.04)		.57
Gender (women)	0.62 (–1.08 to 2.31)		.48	0.78 (–2.58 to 4.13)		.65	0.32 (–0.72 to 1.37)		.55
Platform (Android)	1.8 (–0.34 to 3.26)		.02^d^	2.71 (–0.01 to 5.41)		.05	.69 (–0.25 to 1.63)		.16
Diagnosis (MDD)	10.58 (9.04 to 12.12)		<.001^c^	—^f^		—	—		—

a MDD: major depressive disorder.

b HC: healthy control.

c *P*<.001

d *P<*.05

e PA: physical activity.

f Not available.

Exploratory analyses of associations between PA levels and individual symptoms revealed substantial heterogeneity in effect sizes, with the strongest associations observed for irritability (BDI 11), somatic preoccupation (BDI 20), and loss of libido (BDI 21; Multimedia Appendix 1).

A lifetime diagnosis of MDD was consistently associated with higher depressive symptom severity across all models. Differences between smartphone platforms (Android vs iOS) emerged only in models using weekly time frames.

In the MDD group, the medication index did not significantly influence depressive symptom severity (*β*=0.0198, SE=0.0636; *t*_16682.8_=0.312; *P*=.76), indicating that psychotropic medication use was not a major confounding factor in the observed associations.

### Variability in PA

#### Weekly PA Level Variability

Analysis of the variability of different PA levels reached per week (range 1‐3) revealed significant differences between patients with MDD and HCs (β=–0.75, SE=0.23; *P*=.001). Patients with MDD showed less variability, with 40.9% (74/181) remaining at the same PA level throughout the week compared to 28.2% (55/195) of HCs. A higher proportion of HCs (54/195, 27.5%) compared to patients with MDD (31/181, 17.2%) showed high flexibility by moving through all PA levels within 1 week. The supplementary material provides an additional approach to assess variability, showing violin plots of the SD of PA for MDD and HCs (Multimedia Appendix 1).

#### Daily PA Level Variability

**Figure 2.**
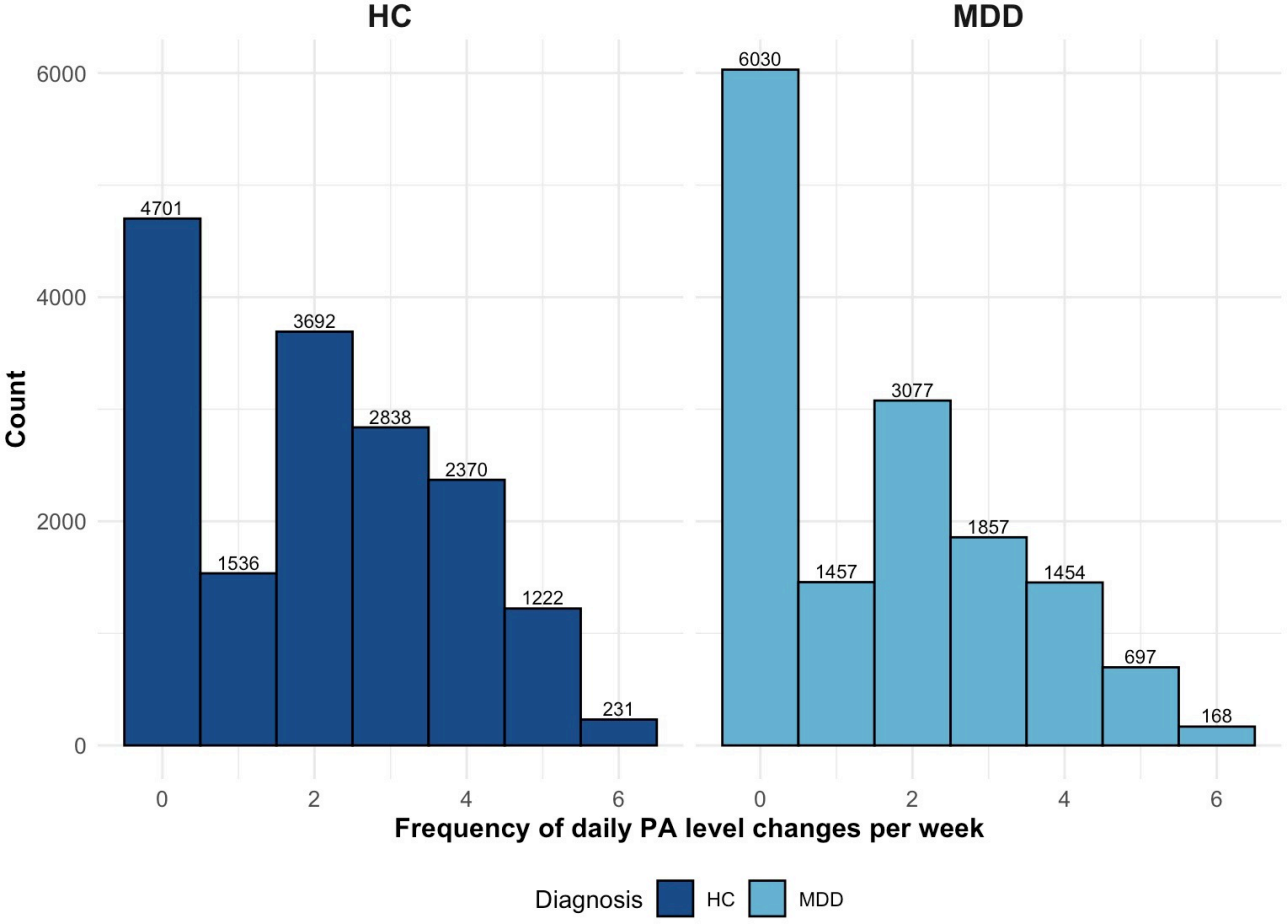
Histogram of daily physical activity level variability per week by diagnosis. HC: healthy control; MDD: major depressive disorder; PA: physical activity.

The variability index, representing fluctuations in daily step counts per week (ranging from no shifts to daily shifts), also showed significant group differences (β=–0.36, SE=0.12; *P*=.003). Patients with MDD were more likely to remain at the same PA level throughout the entire week (37/181, 20.5%) or to experience minimal daily changes (eg, 1 change per week: 15/181, 8.2%) compared to HCs (29/195, 14.9%; 11/195, 5.6%). HCs more frequently showed higher day-to-day variability, with 14.9% (29/195) exhibiting 3 to 5 daily shifts compared to 9.1% (17/181) of patients with MDD, and only 1 (0.3%) HC participant showed daily shifts across all PA levels, compared to none in the MDD group. Figure 2 illustrates the frequency of day-to-day PA level changes across all observation weeks included in the study.

## Discussion

### Principal Findings

This naturalistic study investigated the association between depressive symptom severity and PA as measured by smartphone-based step counts collected via the ReMAP app. Higher PA levels were consistently associated with lower BDI scores across the full sample and within both MDD and HC subgroups. In addition, patients with MDD engaged in less PA compared to HCs and showed reduced day-to-day variability. Moreover, the total number of steps accumulated during the week emerged as a stronger predictor of depressive symptoms than the average weekly activity level.

The findings of the present study hold several implications for clinical practice and future research. First, our results support the hypothesis of a protective effect of higher levels of PA, including more unintentional movements interwoven in daily life, on depression. Importantly, this is consistent with previous findings from several studies. For example, a large body of evidence from longitudinal studies shows that substantial health benefits can be achieved at PA levels even below public recommendations (for a meta-analysis, see [9].

Furthermore, our finding that even coarse step count measurements from smartphones, without the need for dedicated wearables, are associated with lower depressive symptom scores highlights the real-world applicability and scalability of this BYOD approach. As most individuals carry their smartphones daily, passive step tracking presents a feasible method for capturing daily activity patterns and their protective effects on depression, reinforcing its potential for mental health monitoring and intervention. Several prior studies have consistently shown that taking more steps correlates with a reduction in depressive symptoms [24,27,40-42]. These studies raise public awareness of the importance of PA in reducing the risk of depression, but limitations in the study designs leave important questions unanswered. Thus, PA has either been assessed by self-reported questionnaires or focused on interventions [40,43]. Other studies are limited by the use of a cross-sectional design, small sample sizes, or depressive symptoms being surveyed in the general population without a clinical diagnosis of depression or in specific patient groups (eg, US veterans) [24,27,44-46]. Here, we provide evidence that higher PA levels are positively correlated with a reduction in smartphone-based versions of the BDI, which has been shown to provide valid assessments of depressive symptoms [33], in 185 patients with MDD from ongoing longitudinal psychiatric phenotyping studies. As previous studies that have objectively examined the impact of PA on outcomes in patients with MDD have had sample sizes up to 70 participants, our sample is by far the largest.

Moreover, we confirmed that patients with MDD are less physically active than HCs, which is consistent with previous research. Specifically, up to 86% of individuals with MDD do not meet PA guidelines and have decreased PA (standardized mean difference=–0.25, 95% CI –0.03 to 0.15) and increased sedentary behavior (standardized mean difference=0.09, 95% CI=0.01 to 0.18) compared to HCs [47].

### Clinical Implications

Given the limited innovation in treatment and the high nonresponse rates, improving care for depression remains a major challenge. PA, which has proven benefits for both prevention and symptom reduction with minimal side effects, is still difficult to integrate into patients’ daily lives [6,9,48]. In this regard, previous studies have identified depressive symptoms such as loss of interest or pleasure in activities, fatigue, or psychomotor retardation as relevant barriers to engaging in PA [49,50]. Consistent with prior research, our findings indicate that patients with MDD, averaging approximately 4000 steps daily, fall markedly below the established sedentary threshold of 5000 steps per day [24] and average about 1000 steps per day fewer than HCs. As body-tracking devices and smartphone apps have become ubiquitous among us, monitoring and goal-setting for daily steps offer tremendous opportunities for psychiatric clinical practice to overcome some of the common barriers to PA experienced by patients with MDD. For example, a meta-analysis of randomized controlled trials showed a positive effect of mobile apps or activity trackers on PA with any comparison corresponding to 1850 steps per day [51]. The apps and trackers seem to work best when complemented with personalization or text messaging. In this context, personal smartphones emerge as a practical and widely accessible means to facilitate PA promotion.

However, before any recommendations regarding the number of daily steps for individual patients can be made, it is imperative to establish the appropriate threshold. Notably, results from population-based electronic health records, some linked to participants’ wearable devices, suggest that 8200 steps per day may be associated with protection against common diseases, including depression [41]. Ramsey and colleagues recently demonstrated that an increase in steps per day was associated with improvement in depressive symptoms in a longitudinal study with moderate-to-severe MDD over a 24-month follow-up period, suggesting that a 6500-step-per-day increment from the sample mean would result in a clinically meaningful reduction of 5 points in the Patient Health Questionnaire (PHQ-9) [24].

It should be noted, however, that merely the number of steps is not sufficient to achieve significant benefits from PA in patients with MDD. For example, some patients may feel overwhelmed by an ambitious daily step goal and may have difficulty maintaining it in their daily routine. Moreover, it is not yet known how long it should take to gradually increase the number of steps until the final target is reached, or whether “rest days” of low-intensity exercise at regular intervals might have an additional benefit.

Our findings highlight that patients with MDD exhibit less variability in PA levels compared to HCs, which may reflect a more rigid or constrained activity pattern. Notably, our analysis revealed that the total number of steps accumulated during the week was a stronger predictor of depressive symptoms than average weekly activity levels, suggesting that fluctuations in PA patterns, such as balancing lower activity on some days with higher activity on others, may still have a protective effect against depression severity. These findings underscore the significance of personalized and adaptable activity patterns, as opposed to rigid daily targets, when assessing PA in the context of depression. Further research is needed to develop evidence-based PA guidelines for patients with MDD that account for individual performance levels and allow for adaptive pacing. These guidelines have the potential to encourage more widespread and sustainable use of PA as part of a comprehensive approach to depression management. Beyond behavioral patterns, the mechanistic basis for PA’s antidepressant effects is increasingly understood. Preclinical studies indicate that PA and antidepressant medications act on overlapping biological pathways and may exert complementary effects on depressive-like behavior [52]. In addition, PA positively affects psychosocial factors such as self-esteem, social support, and self-efficacy, which may synergize with biological mechanisms to enhance its antidepressant effects [11].

Importantly, PA not only improves mental health outcomes but also addresses key pathophysiological mechanisms shared between MDD and common somatic comorbidities. These include chronic low-grade inflammation and metabolic dysregulation, which contribute to the elevated cardiovascular risk in this population [53]. Taken together, our results underscore the potential of tailored lifestyle interventions, particularly PA-based programs, to mitigate depressive symptoms and simultaneously reduce cardiometabolic risk. Future studies should further explore such integrative treatment strategies, especially in patients with MDD with complex comorbidity profiles.

### Limitations

The results of this study should be viewed in the context of several limitations. First, information about steps walked is retrieved from Apple HealthKit or Google Fit by using smartphone sensors. Using ReMAP, which has been demonstrated to be a technically feasible tool, assessments of PA and BDI were conducted with high temporal resolution with both active and passive data collection, thus delivering data of high ecological validity [32]. The use of mobile devices, particularly smartphones, for remote monitoring and data collection has grown rapidly in clinical research, providing valuable insights into the daily lives of patients with MDD [54]. Despite their popularity, research is inconclusive as to whether commercial devices are valid and reliable methods for estimating parameters associated with PA, including steps, which generally tend to be underestimated but still highly correlated with measurements during laboratory-based assessment [28,29]. Importantly, there is still a lack of consistency in published protocols regarding the validity and reliability of mobile apps for measuring PA due to the variety of devices, limited valid comparisons, and study protocols [30,31]. Therefore, optimizing research efforts in this specific area is essential. However, when comparing PA measurement techniques, commercial mobile devices tend to be less accurate. In particular, lower velocities and carrying the smartphone close to the hip, such as in a pants pocket, seem to have a negative impact on reliability [30]. Moreover, considerable heterogeneity in the continuity of data transmission was found: While the continuity of passive data transfer appeared to be excellent for iOS users, a significantly lower rate of passive events per day was observed for Android users [32]. Although some accuracy is lost, smartphone-derived data is very accessible to a large portion of the population and is associated with high levels of adherence.

Second, the moderate-to-strong correspondence between digital and conventional BDI scores observed here supports the validity of the ReMAP BDI, though somewhat lower than in prior controlled validation work [33], likely due to the naturalistic design and narrower symptom range in our cohort. Moreover, it is essential to acknowledge that this study adopted an observational approach, precluding the drawing of causal conclusions. While our findings suggest a robust association between higher step counts and lower depressive symptom severity, the directionality of this relationship remains unclear. It is plausible that individuals with fewer depressive symptoms are more likely to engage in PA, rather than PA directly reducing symptom severity. This bidirectional relationship is supported by our data, which consistently show that participants with lower symptom levels were more active at all time points. Therefore, interpretations regarding the antidepressant effects of PA should be made with caution, and future research using experimental or longitudinal designs is needed to disentangle causal pathways.

Third, our data were limited to step-based measures of PA and therefore did not capture non-stepping activities (eg, swimming and cycling) or allow differentiation between activity types and contexts (eg, daily walking vs structured exercise). As a result, step counts represent overall locomotor activity rather than specific forms or intensities of physical exercise. Future studies integrating multimodal smartphone or wearable data (eg, GPS or heart rate sensors) could provide a more comprehensive understanding of both the type and intensity of PA.

Fourth, no information is available on any technical problems or anomalies that may have occurred during the observation phase, as there was no further mandatory direct contact with the participants.

Fifth, it should also be noted that part of the data collection overlapped with the COVID-19 pandemic, a global event that profoundly affected daily routines, mobility, and mental health. Exploratory analyses (not shown) indicated slightly higher depressive symptom scores during pandemic relaxation periods compared with lockdown phases, particularly among participants with MDD. Nevertheless, the overall negative association between PA and depressive symptoms remained stable across pandemic phases. These findings align with our previous ReMAP COVID-19 study [55] and other reports showing that pandemic-related restrictions were associated with reduced PA and increased depressive and anxiety symptoms [56-62]. The pandemic context should therefore be considered an important external factor when interpreting our results. Moreover, although participants with severe somatic conditions were excluded and BMI did not significantly affect the results, lifestyle factors such as smoking status or alcohol consumption were not available in all cohorts. Residual confounding by these variables cannot be entirely ruled out and should be addressed in future research.

Finally, the effects of PA are not independent of depression treatment, including medication, psychotherapy, or other factors that may affect depressive symptoms. Moreover, although participants with severe somatic conditions were excluded and BMI did not significantly affect the results, lifestyle factors such as smoking status or alcohol consumption were not available in all cohorts. Residual confounding by these variables cannot be entirely ruled out and should be addressed in future research.

### Conclusions

In conclusion, our study indicates a strong link between PA and reduced depressive symptom severity. In a large naturalistic cohort, higher daily step counts were consistently linked to lower depression scores, with cumulative weekly activity emerging as a particularly relevant factor. This suggests that flexible activity patterns, which allow for fluctuations across days, may be as beneficial as consistently high daily PA.

Beyond its role as a protective behavioral factor, this study also demonstrates the feasibility of using smartphone-based tools to collect real-time PA data in patients with MDD. Such data could facilitate the integration of personalized activity goals into everyday life and inform adaptive, patient-centered interventions. This approach holds great promise for application in clinical settings, including as an adjunct to psychotherapy and serving as a behavioral activator, adherence facilitator, or feedback-enhancing tool.

Future research should aim to clarify the biological mechanisms linking PA and depression and develop evidence-based, individualized PA recommendations tailored to the needs of patients with MDD. These efforts may ultimately contribute to improved treatment outcomes and promote sustainable lifestyle changes in the management of depression.

## Supplementary material

10.2196/81120 Multimedia Appendix 1 Distribution of daily step variability per person and Manhattan plots of associations between step count levels and individual Beck Depression Inventory items in major depressive disorder and healthy control samples.

## Abbreviations

BDI: Beck Depression Inventory
BYOD: bring-your-own-device
*DSM-IV*: *Diagnostic and Statistical Manual of Mental Disorders* (Fourth Edition)
HC: healthy control
ICC: intraclass correlation coefficient
MDD: major depressive disorder
PA: physical activity
PHQ-9: 9-item Patient Health Questionnaire
ReMAP: Remote Monitoring Application in Psychiatry
SCID: structured clinical interview for *Diagnostic and Statistical Manual of Mental Disorders* (Fourth Edition)
