# Supplementary material for: Association of Daily Step Count With Depressive Symptoms in Patients With Major Depressive Disorder Using a Smartphone App (ReMAP): Longitudinal Study
Source: JMIR Ment Health. 2026 Feb 10;13:e81120. doi: 10.2196/81120 (PMC12894579; doi:10.2196/81120)

Supplements Extra

Supplementary Extra Figure 1: Distribution of Daily Step Variability (SD) per Person

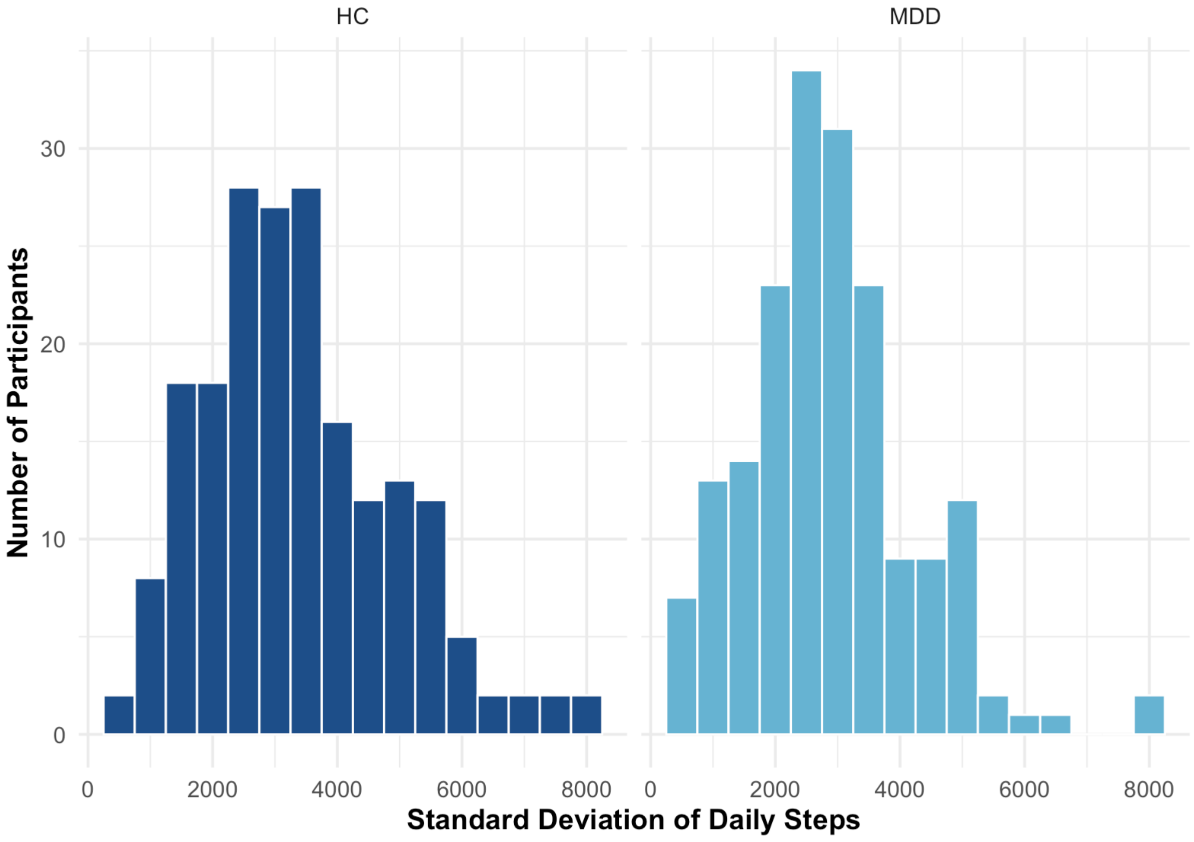

Supplementary Extra Figure 2: Manhattan Plot of Associations Between Step Count Levels and Individual BDI Items; -$\log_{10}$ p-values Displayed (MDD Sample)

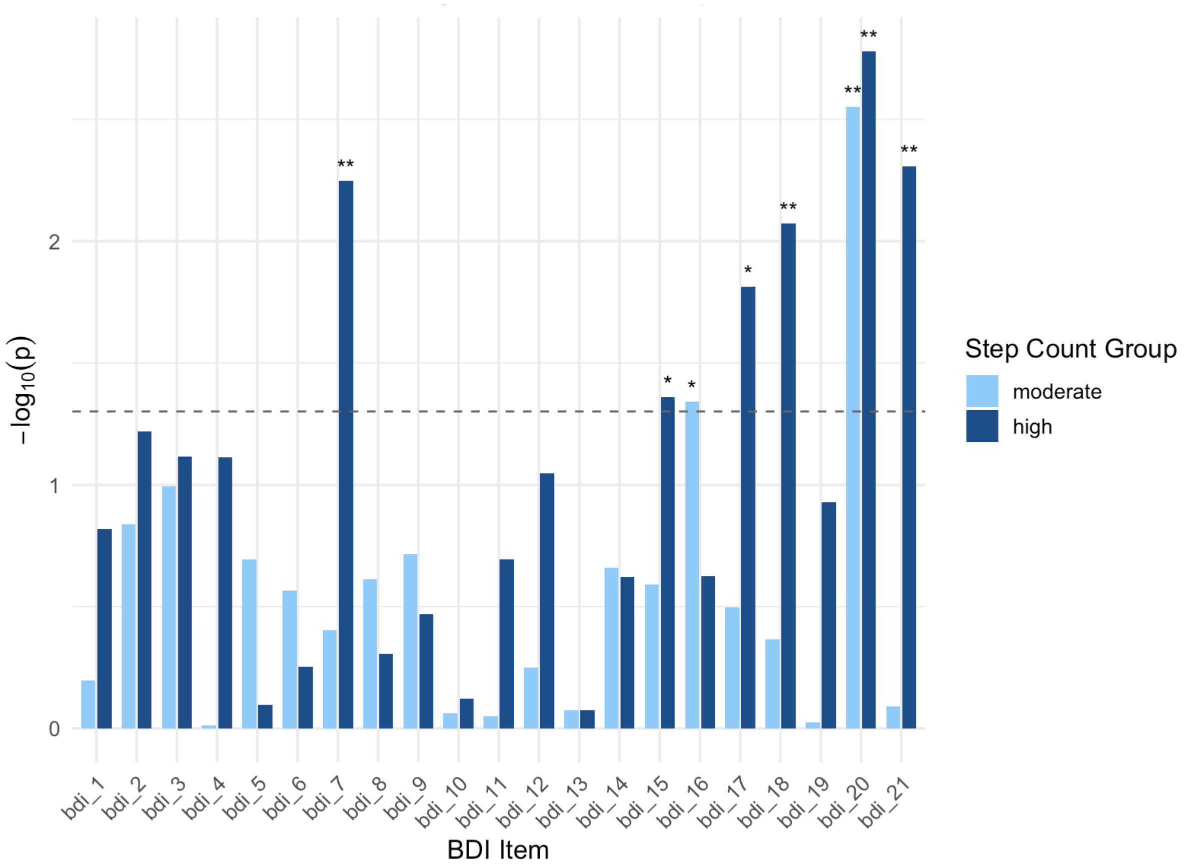

Supplementary Extra Figure 3: Manhattan Plot of Associations Between Step Count Levels and Individual BDI Items; -$\log_{10}$ p-values Displayed (HC Sample)

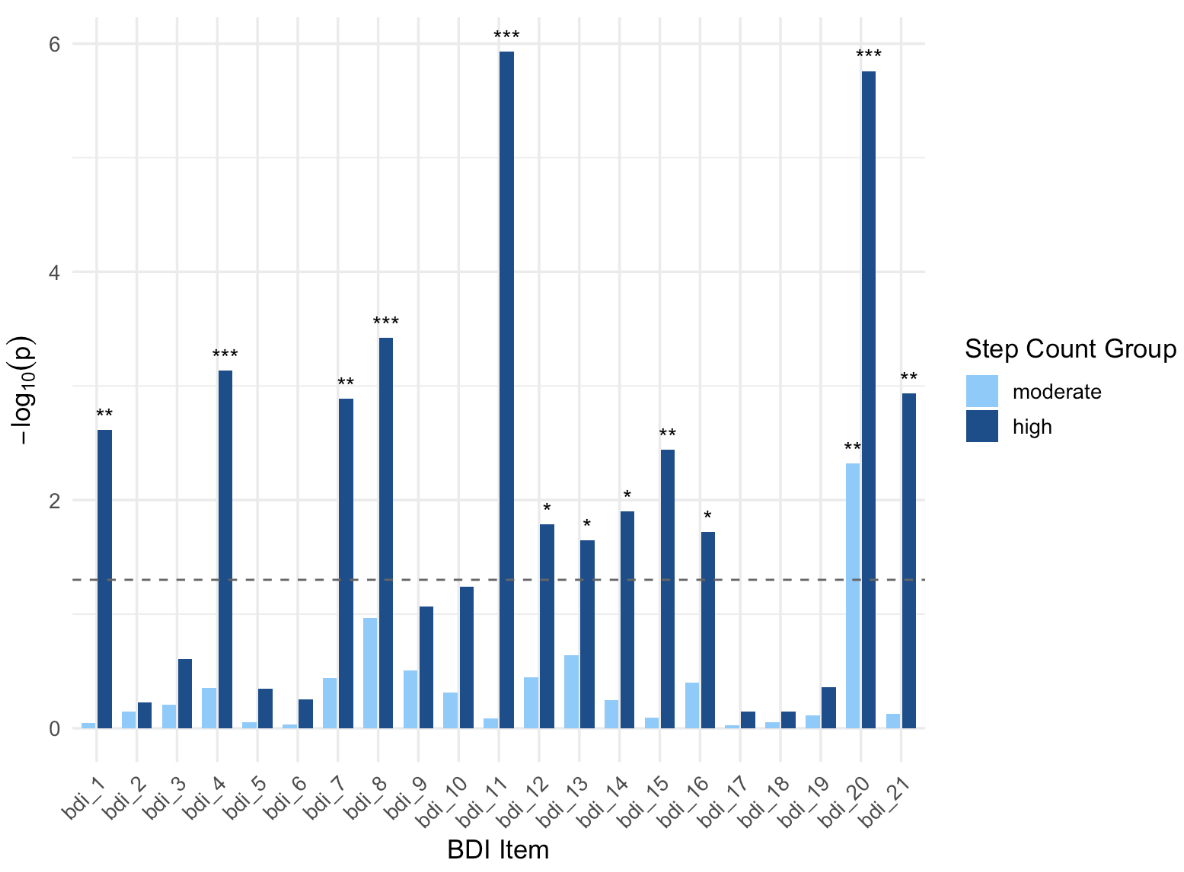

Supplement: Multimedia Appendix 1 [file mental-v13-e81120-s001.pdf]
